# Supplementary material for: Modelling Skylarks (Alauda arvensis) to Predict Impacts of Changes in Land Management and Policy: Development and Testing of an Agent-Based Model
Source: PLoS One. 2013 Jun 6;8(6):e65803. doi: 10.1371/journal.pone.0065803 (PMC3675089; doi:10.1371/journal.pone.0065803)
Supplement: Supporting Information S4 — The skylark ODdox as a zipped archive. (ZIP) [file pone.0065803.s004.zip › Skylark_ODdox/configurator_8cpp.html]

ALMaSS Skylark ODdox: configurator.cpp File Reference


ALMaSS Skylark ODdox
 2.0


- Main Page
- Related Pages
- Classes
- Files

- File List
- File Members

Macros |
Functions |
Variables

configurator.cpp File Reference

`#include <stdio.h>`  
`#include <iostream>`  
`#include <fstream>`  
`#include <stdlib.h>`  
`#include <string.h>`  
`#include "../BatchALMaSS/ALMaSS_Setup.h"`  
`#include "configurator.h"`  
`#include "maperrormsg.h"`  
`#include <crtdbg.h>`

|  |  |
| --- | --- |
| Macros | |
| #define | \_CRT\_SECURE\_NO\_DEPRECATE |
| #define | DEBUG\_NEW   new(\_NORMAL\_BLOCK, \_\_FILE\_\_, \_\_LINE\_\_) |

|  |  |
| --- | --- |
| Functions | |
| void | FloatToDouble (double &, float) |

|  |  |
| --- | --- |
| Variables | |
| static const char \* | CfgSecureStrings [] |
| static const char \* | CfgTypeStrings [] |
| class Configurator \* | g\_cfg = NULL |
| static CfgBool | l\_cfg\_public\_exit\_on\_set ("CFG\_PUBLIC\_EXIT\_ON\_SET", CFG\_CUSTOM, true) |
| static CfgBool | l\_cfg\_public\_warn\_on\_set ("CFG\_PUBLIC\_WARN\_ON\_SET", CFG\_CUSTOM, true) |

---

## Macro Definition Documentation

#define \_CRT\_SECURE\_NO\_DEPRECATE

#define DEBUG\_NEW   new(\_NORMAL\_BLOCK, \_\_FILE\_\_, \_\_LINE\_\_)

---

## Function Documentation

|  |  |  |  |
| --- | --- | --- | --- |
| void FloatToDouble | ( | double & | , |
|  |  | float |  |
|  | ) |  |  |

Referenced by CropData::CropData(), VegElement::ReadBugPercentageFile(), Configurator::SetCfgFloat(), and Weather::Weather().

{

char \* num = 0;

num = new char[\_CVTBUFSIZE];

errno\_t err = \_gcvt\_s(num, \_CVTBUFSIZE,f,8);

if (err!=0) {

assert(0);

}

d = atof(num);

delete [] num;

}

---

## Variable Documentation

const char\* CfgSecureStrings[]

static

**Initial value:**

{

"CFG\_CUSTOM",

"CFG\_PUBLIC",

"CFG\_PRIVATE"

}

Referenced by Configurator::DumpSymbols().

const char\* CfgTypeStrings[]

static

**Initial value:**

{

"\*none\*",

"int",

"double",

"bool",

"string"

}

Referenced by Configurator::DumpSymbols(), and Configurator::ShowIdType().

class Configurator\* g\_cfg = NULL

Referenced by CfgBase::CfgBase(), CloseDownSim(), Landscape::DumpAllSymbolsAndExit(), Landscape::DumpPublicSymbols(), Landscape::Landscape(), and Landscape::ReadSymbols().

CfgBool l\_cfg\_public\_exit\_on\_set("CFG\_PUBLIC\_EXIT\_ON\_SET", CFG\_CUSTOM, true)

static

Referenced by Configurator::SetCfgGatekeeper().

CfgBool l\_cfg\_public\_warn\_on\_set("CFG\_PUBLIC\_WARN\_ON\_SET", CFG\_CUSTOM, true)

static

Referenced by Configurator::SetCfgGatekeeper().


- CJT
- MSVC
- ALMaSS Working Source
- Landscape
- configurator.cpp
- Generated on Thu Jan 10 2013 13:15:35 for ALMaSS Skylark ODdox by
   1.8.1.1
